# Downregulation of the neuronal opioid gene expression concomitantly with neuronal decline in dorsolateral prefrontal cortex of human alcoholics

**DOI:** 10.1038/s41398-017-0075-5

**Published:** 2018-06-20

**Authors:** Igor Bazov, Daniil Sarkisyan, Olga Kononenko, Hiroyuki Watanabe, Victor M. Karpyak, Tatiana Yakovleva, Georgy Bakalkin

**Affiliations:** 1 Division of Biological Research on Drug Dependence, Department of Pharmaceutical Biosciences, Uppsala University, SE-751 24 Uppsala, Sweden; 2 Department of Psychiatry and Psychology, Mayo Clinic College of Medicine, Rochester, MN 55905 USA

## Abstract

Molecular changes in cortical areas of addicted brain may underlie cognitive impairment and loss of control over intake of addictive substances and alcohol. Prodynorphin (PDYN) gives rise to dynorphin (DYNs) opioid peptides which target kappa-opioid receptor (KOR). DYNs mediate alcohol-induced impairment of learning and memory, while KOR antagonists block excessive, compulsive-like drug and alcohol self-administration in animal models. In human brain, the DYN/KOR system may undergo adaptive changes, which along with neuronal loss, may contribute to alcohol-associated cognitive deficit. We addressed this hypothesis by comparing the expression levels and co-expression (transcriptionally coordinated) patterns of *PDYN* and KOR (*OPRK1*) genes in dorsolateral prefrontal cortex (dlPFC) between human alcoholics and controls. Postmortem brain specimens of 53 alcoholics and 55 controls were analyzed. *PDYN* was found to be downregulated in dlPFC of alcoholics, while *OPRK1* transcription was not altered. *PDYN* downregulation was confined to subgroup of subjects carrying C, a high-risk allele of *PDYN* promoter SNP rs1997794 associated with alcoholism. Changes in *PDYN* expression did not depend on the decline in neuronal proportion in alcoholics, and thereby may be attributed to transcriptional adaptations in alcoholic brain. Absolute expression levels of *PDYN* were lower compared to those of *OPRK1*, suggesting that *PDYN* expression is a limiting factor in the DYN/KOR signaling, and that the *PDYN* downregulation diminishes efficacy of DYN/KOR signaling in dlPFC of human alcoholics. The overall outcome of the DYN/KOR downregulation may be disinhibition of neurotransmission, which when overactivated could contribute to formation of alcohol-related behavior.

## Introduction

Alcohol consumed in moderate and large amounts causes acute and delayed impairments in cognitive and executive functions that guide complex behavior through planning, decision-making, and response control^1–6^. Total alcohol consumption and binge drinking patterns are risk factors for dementia^7–9^. Alcohol-induced cognitive deficit is a factor underlying the habitual drug seeking and taking that characterize addiction and dependence^10,11^.

The mechanism of alcohol-induced cognitive impairments remains unknown but may involve neurodegeneration and aberrant neurotransmission. Several studies indicate that cognitive effects of alcohol may be mediated through dysregulation of the dynorphin/κ-opioid receptor (DYN/KOR) system in the prefrontal cortex (PFC) and hippocampus^12–16^. DYN opioid peptides have been implicated in cognitive decline^17–22^. Administration of synthetic DYN into dorsal hippocampus impairs spatial learning in rats^17^. DYNs contribute to age-related and stress-induced deficits in learning and memory^18–20^. In elderly humans, prodynorphin (*PDYN*) gene polymorphisms play a role in memory function^22^. In individuals with Alzheimer’s disease, DYN is elevated in PFC and this increase correlates with neuropathological lesions^21^.

A role of the DYN/KOR system in impairment of spatial learning and memory was identified in a rat model of cognitive deficit induced by alcohol binge drinking^16,23,24^. Selective KOR antagonist nor-binaltorphimine normalized animal performance in spatial learning and memory tasks possibly by reversion of ethanol-induced elevation in glutamate overflow.

Molecular and cellular allostatic changes in addicted brain may underlie addictive behavior and associated phenomena including impairment of cognitive functions^25^. Changes in the DYN/KOR system may be involved in allostatic processes in the addicted brain as demonstrated in animal studies^16,26–34^. The aim of the present study was to examine whether the DYN/KOR system undergoes adaptive changes at the transcriptional level in dorsolateral prefrontal cortex (dlPFC) in human alcoholics. We previously conducted a pilot analysis of a limited number of human alcoholics and controls that revealed no differences in the expression of the *PDYN* gene in the orbitofrontal cortex (OFC) and *OPRK1* gene (opioid receptor kappa 1 gene encoding KOR protein) in dlPFC, and elevation with a borderline significance of *PDYN* and KOR mRNA levels in dlPFC and OFC, respectively, of alcoholics^35^. The number of subjects is a critical factor in molecular analysis of human brain. Reasoning that our previous study was possibly underpowered, in the present work the number of subjects was increased nearly 4-fold and more stringent statistical analysis was applied.

Analysis of absolute expression levels revealed a co-regulated (transcriptionally coordinated) pattern of *PDYN* (prodynorphin gene encoding precursor of DYN peptides) and *OPRK1* expression in human nucleus accumbens (NAc)^36^. This pattern was significantly different between alcoholics and controls. To assess whether the *PDYN* and *OPRK1* genes are co-regulated in dlPFC, and whether this co-regulation is altered in addicted dlPFC, we compared *PDYN* and *OPRK1* co-expression along with average expression levels of these genes in dlPFC between alcoholics and controls.

A number of neurons is markedly reduced in alcoholics in dlPFC as demonstrated in early postmortem morphological studies^37,38^ and confirmed by analyses of neuronal proportion quantified from epigenome-wide DNA methylation profiles and expression of neuronal marker (*RBFOX3* encoding NeuN protein)^39^. To attribute potential expression changes to transcriptional events or changes in cell composition in alcoholics, we examined whether *PDYN* and KOR (*OPRK1*) mRNAs are expressed strictly in neurons in human brain and then analyzed effects of the decline in neuronal proportion and *RBFOX3* expression on *PDYN* and *OPRK1* mRNA alterations in alcoholic brain. Effects of *PDYN* and *OPRK1* single-nucleotide polymorphisms (SNPs) strongly associated with alcoholism on expression of both genes were also studied.

## Materials and methods

### Human samples

Human frozen brain tissues were collected at the New South Wales Brain Tissue Resource Centre (NSW BTRC), University of Sydney, Australia (https://sydney.edu.au/medicine/pathology/btrc/; see Table 1 for short summary and Supplementary Table 1 for detailed information). Tissue samples from 55 control and 53 alcoholic subjects, all males of European descent, were analyzed. Alcoholics were the subjects that met Diagnostic and Statistical Manual for Mental Disorders, 4th edition (DSM-IV) criteria for Alcohol Abuse or Alcohol Dependence and consumed 206 ± 20 g (mean ± S.E.M.) of ethanol per day in average for the majority of their adult lives^40^. Controls had either abstained from alcohol completely or were social drinkers who consumed 17 ± 3 g of ethanol per day on average. Methods used to classify alcoholics were described previously^40,41^. Cases with a history of polydrug abuse (with evidence that the individual abused other drugs such as cocaine or heroin) or with medical complications such as Wernicke–Korsakoff syndrome or alcoholic cases with concomitant diseases were excluded. Cases with a history of cerebral infarction, head injury, or neurodegenerative disease (e.g., Alzheimer’s disease) were also excluded. dlPFC samples were dissected from superior frontal gyrus/Brodmann area 9. Informed written consent for autopsy was obtained from the next-of-kin and collection was approved by the Human Research Ethics Committees of the Sydney Local Health District (X15-0199) and the University of Sydney. The study was approved by the Swedish Central Ethical Review Board. Smoking status information was available for 94% of subjects (Supplementary Table 1; “ex-smokers” were grouped with “non-smokers”).

**Table 1:** Summary of demographic data and tissue characteristics of human subjects (for details, see Supplementary Table 1)

	*N*	Age	PMI	pH	RQI	Neuronal proportion^a^
*Analysis of gene expression*						
Controls	55	54.7 ± 9.1	30.0 ± 12.5	6.6 ± 0.2	8.1 ± 1.0	
Alcoholics	53	56.2 ± 8.9	35.8 ± 15.3	6.5 ± 0.3	7.4 ± 1.3	
*P* -value		n.s.	0.033	0.015	0.002	
*Analysis of neuronal proportion*						
Controls	35	54.9 ± 9.6	28.5 ± 13.6	6.6 ± 0.2	8.1 ± 1.1	0.24 ± 0.04
Alcoholics	30	58.6 ± 9.5	31.9 ± 15.3	6.5 ± 0.3	7.4 ± 1.3	0.21 ± 0.07
*P* -value		n.s.	n.s.	n.s.	0.017	0.033

Values are means ± SD for each cohort; *SD* standard deviation, *N* a number of subjects, *Age* age in years, *PMI* post-mortal interval in hours, *pH* brain pH, *RQI* RNA quality indicator

Unpaired *t*-test was used to calculate *P-*values. *n.s.* not significant

^a^Data are taken from our previous paper^39^. Neuronal proportion was quantified from epigenome-wide DNA methylation profiles

### RNA purification

Total RNA was purified using RNeasy Lipid Tissue Mini Kit (Qiagen) and treated with RNase-free DNase I (Qiagen) on column, according to the manufacturer’s recommendations. RNA concentrations and 260/280 and 260/230 ratios were measured with a Nanodrop. RNA quality indicator (RQI) was measured using Bio-Rad Experion (Bio-Rad) with Eukaryote Total RNA StdSens assay, according to the manufacturer’s protocol. 500 ng RNA were reverse-transcribed to cDNA in duplicates with the High-Capacity RNA-to-cDNA kit (Applied Biosystems), according to the manufacturer’s recommendations.

### Gene expression analysis

TaqMan assays (Applied Biosystems) for *GFAP* (Hs00909233_m1), *OPRK1* (Hs00175127_m1), *PDYN* (Hs00225770_m1), *POLR2A* (Hs00172187_m1), *RBFOX3* (Hs01370653_m1), and *RPLP0* (Hs99999902_m1) were used. cDNAs were mixed with TaqMan assay and iTaq Universal Probes supermix (Applied Biosystems) for qPCR with a CFX96 Real-Time Detection System (Bio-Rad), according to the manufacturer’s instructions. Levels of each gene of interest mRNA were normalized to geometric mean of expression levels of two control genes *POLR2A* and *RPLP0* selected by geNORM program (https://genorm.cmgg.be/)^42^ (see also our studies^35,43,44^). In each experiment, internal control gene-stability measure *M*^42^ was controlled for and did not exceed the limit of 0.5.

### Radioimmunoassay (RIA)

The procedure has been described elsewhere^45,46^. Briefly, 1 M hot acetic acid was added to finely powdered frozen brain tissues, and samples were boiled for 5 min, ultrasonicated and centrifuged. Tissue extracts were run through SP-Sephadex ion exchange C-25 column, and peptides were eluted and analyzed by RIA. Anti-Dyn B antiserum showed 100% molar cross-reactivity with Big DYN, which consists of Dyn A and Dyn B sequences, 0.8% molar cross-reactivity with Leu-morphine (29 amino acid C-terminally extended Dyn B), and <0.1% molar cross-reactivity with Dyn A (1–17), Dyn A (1–8), *α*-neoendorphin, and Leu-enkephalin^47^.

### Isolation of cell nuclei

Tissue samples were Dounce homogenized in the lysis buffer (0.32 M sucrose; 5 mM CaCl_2_; 3 mM magnesium acetate; 0.1 mM EDTA; 10 mM Tris-HCl, pH 8.0; 0.1% Triton X-100; 1 mM DTT). Homogenized samples were gently suspended in sucrose solution (1.7 M sucrose; 3 mM magnesium acetate; 1 mM DTT; 10 mM Tris-HCl, pH 8.0), and layered onto a sucrose cushion. Ultracentrifugation was carried out at 30,000 × *g* for 2.5 h at 4 °C (Beckman; L8-70 M; SW28 swing bucket rotor). After centrifugation, the supernatant was removed by aspiration. Nuclei pellets resuspended in PBS were filtered through a 40 μm Nitex mesh to remove remaining clumps.

### Flow cytometry

Neuronal nuclei were isolated by fluorescence-activated nuclei sorting (FANS) after labeling with neuron-specific monoclonal antibody against NeuN (MAB377, Millipore). NeuN antibodies conjugated with mouse IgG labeling reagent (Alexa 488, Molecular Probes) were incubated with nuclear suspension for 30 min in the dark and directly sorted in the RLT lysis buffer (Qiagen). FANS was performed using a FACSAria III cell sorter (BD BioSciences), nuclei were pelleted by centrifugation at 3000 x *g* for 5 min at 4 °C, and stored at −80 °C. To ensure sorting of single but not aggregated nuclei preparations were stained with Hoesch dye, and a gate was set to isolate singlets only that were readily discerned from doublets, triplets, and higher-order aggregates based on their fluorescence intensity. The purity of neuronal nuclei was confirmed by FANS analysis of the sorted preparations.

### Droplet digital PCR

The assay was described elsewhere^48^. Total RNA from FANS-sorted nuclei was purified using RNeasy Plus Mini kit (Qiagen) and 15–60 ng RNA were reverse-transcribed with the High-Capacity RNA-to-cDNA kit (Applied Biosystems), according to the manufacturer’s recommendations. cDNAs were mixed with TaqMan assay, ddPCR Supermix for Probes (Bio-Rad) and Droplet Generation Oil (Bio-Rad), partitioned into 14,000–17,000 droplets in QX200 Droplet Generator and used for PCR with T100 Thermal Cycler (Bio-Rad), according to the manufacturer’s instructions. The fluorescence intensity of the droplets was measured using the QX200 Droplet Reader (Bio-Rad). The data analysis was performed with QuantaSoft droplet reader software (Bio-Rad). mRNA amount was calculated using the Poisson statistics^49^. The absolute transcript levels were expressed in mRNA copies per ng of total RNA. Correlation between *PDYN* expression levels obtained using ddPCR and qRT-PCR was positive and significant (Pearson *R* = 0.9, *P* = 0.0004) (Supplementary Fig. 1).

### Genotyping

SNPs were determined by SNP&SEQ Technology Platform at Uppsala University using Illumina HumanOmni5Exome-4v1 beadchip.

### Computation of neuronal proportions

Genome-wide DNA methylation data for 482,421 CpGs in DNA from total tissue was profiled by SNP&SEQ Technology Platform at Uppsala University using Illumina Infinium HumanMethylation450 beadchip and processed using R package Cell EpigenoType-Specific *CETS* mapper. *CETS* predicts neuronal proportions from methylation levels of the top 10,000 marker CpGs, which demonstrated the most significant methylation differences between neuronal and non-neuronal DNA^39,50^.

### Statistical analysis

Statistical analysis was performed using R version 3.3.2 (https://www.R-project.org/). Statistica 13 (StatSoft Scandinavia, Sweden) was used for analysis of absolute expression levels by unpaired Student’s *t*-test and for sample size calculation of one-way ANOVA by statistical power analysis with target Type I error *α* = 0.05 and statistical power *β* = 0.8. For analysis of gene expression linear regression model adjusting for age, PMI, brain pH, RQI, and alcoholism followed by post hoc Tukey HSD test on least squares means was performed using *car* and *lsmeans* packages. Gene expression was adjusted for *RBFOX3* mRNA levels as a surrogate measure of neuronal proportion and/or activity; it was further adjusted for neuronal proportion instead of *RBFOX3* mRNA levels. An interaction effect between alcoholism and KOR (*OPRK1*) mRNA levels was also considered. Smoking, mean and total alcohol consumption, DSM-V severity of alcohol use disorder and four alcoholism-associated SNPs were added to the model one at a time to test if they can account for gene expression variance. Overly influential points with Cook’s distance ≥1.0 were removed from analysis of the above models^51^. One subject was excluded as a biological outlier; and two subjects were excluded as statistical outliers. *R* package *effects* was used to construct effect displays (component and residual plots). Bootstrapped *P*-values, and bias-corrected and accelerated bootstrap percentile 95% confidence intervals (CI) for regression coefficients, both of which do not require the assumption of normality, were estimated using *car* package with *R* = 5 × 10^5^ resampled cases^52^. Script to analyze data and generate figures is written using publicly available software R and is available from authors upon request. A significance level of *P* < 0.05 was accepted as statistically significant and all tests were two-tailed.

## Results

Fifty-three DSM-IV alcoholic and 55 control subjects were analyzed in the study. Effects of alcoholism on the whole tissue levels of *PDYN* and *OPRK1* mRNAs in dlPFC were examined after adjusting for demographical data and tissue characteristics including age, PMI, brain pH, and RQI (Table 1). We and others demonstrated that the number of neurons in dlPFC is markedly reduced in alcoholics^37–39^. Tissue expression levels may differ between the subject groups due to changes in cell composition if genes of interest are transcribed in specific cell types. Therefore, neuronal proportion computed by using genome-wide DNA methylation data, and mRNA levels of neuronal marker *RBFOX3*, which correlates with neuronal proportion, were included as confounding factors in the analysis (for details, see ref. ^39^ and Table 1).

### *PDYN* and *OPRK1* gene expression in human dlPFC: absolute levels and cell-type specificity

**Fig. 1:**
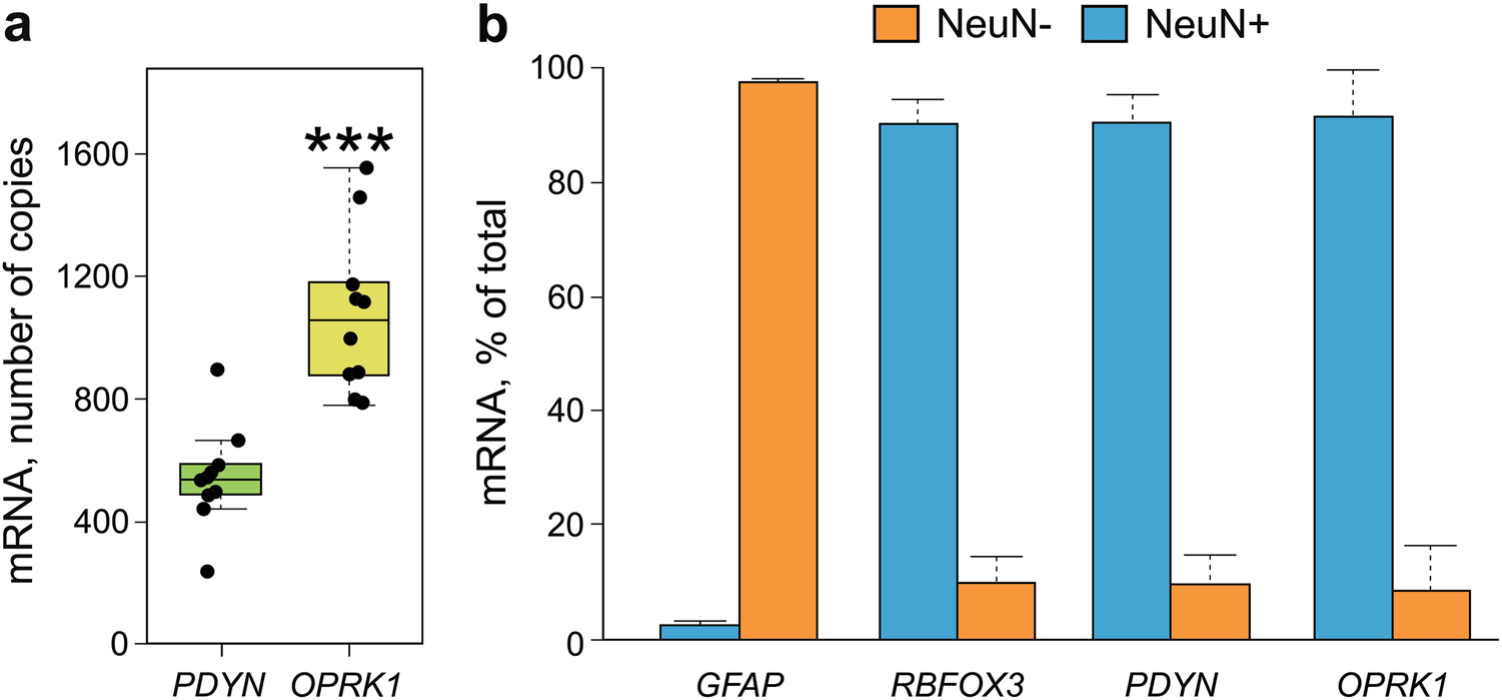
Expression of the *PDYN* and KOR (*OPRK1*) genes in human dlPFC. **a** Absolute levels of *PDYN* and *OPRK1* mRNAs measured by ddPCR using total tissue RNA (*n* = 10 control subjects), presented as a number of mRNA copies/ng of total RNA. Student’s *t*-test, ***P < 0.001. In box plots, middle line is the median, box spans the interquartile range (IQR), and whiskers extend 1.5 × IQR from box limits. **b** Neuronal expression of *PDYN* and *OPRK1* genes in dlPFC. Levels of *RBFOX3* (neuronal marker), *PDYN*, and *OPRK1* mRNAs are high in neuronal nuclei (NeuN+), while those of *GFAP* (astrocyte marker) in non-neuronal (NeuN-) nuclei. Bar graphs show average mRNA amount ± S.E.M. in NeuN+ and NeuN- fractions as % of total amount of these mRNAs. Nuclei were isolated by FANS individually from dlPFC tissue of *n* = 3 (used for analysis of *PDYN*, *RBFOX3*, and *GFAP* mRNAs) or *n* = 2 (used for analysis of *OPRK1* mRNA) subjects. mRNA levels were analyzed by ddPCR and normalized to total RNA content

Receptor activation depends on concentration of its ligands, which may be produced in shortage or in excess relative to the receptor^53^. We examined whether a limiting factor in the DYN/KOR signaling in dlPFC may be defined at the level of *PDYN* or KOR (*OPRK1*) gene transcription by comparing absolute levels of *PDYN* and *OPRK1* mRNAs by ddPCR using cDNA prepared from total dlPFC tissue samples from control subjects (Fig. 1a). The *PDYN* mRNA levels were significantly 2-fold lower compared to those of *OPRK1* levels (*t*_(18)_ = −5.3864, *P* = 4.1 × 10^−5^; Fig. 1a). Considering rapid DYN degradation and relatively large extracellular volume in which the peptides are diluted, the concentration of DYNs in the vicinity of the receptor molecules should be low resulting in the receptors' underactivation. Thus, transcriptional processes may already establish proportion in the expression of *PDYN* and KOR (*OPRK1*) genes rendering the DYN production to be a limiting factor in the DYN/KOR signaling in dlPFC.

Expression levels of *PDYN* and KOR (*OPRK1*) in dlPFC may depend on proportion of cell type(s), specifically neurons, transcribing these genes in the tissue. To confirm neuronal expression of *PDYN* and *OPRK1* in human dlPFC, we analyzed levels of *PDYN* and KOR (*OPRK1*) mRNAs by ddPCR in neuronal and non-neuronal cell nuclei isolated from this tissue samples by FANS with antibodies against NeuN, the neuronal marker transcribed from the *RBFOX3* gene (Fig. 1b). mRNAs of *RBFOX3* and glial fibrillary acidic protein (GFAP) genes, the neuronal and astrocyte markers analyzed as positive and negative controls were localized in neuronal and non-neuronal nuclei, respectively. Both *PDYN* and *OPRK1* mRNAs were highly enriched, approx. 10-fold in neuronal compared to non-neuronal nuclei (Fig. 1b). Neuronal proportion and mRNA levels of neuronal marker NeuN (*RBFOX3*) were then included as covariates in statistical models for comparison of *PDYN* and *OPRK1* tissue expression levels between pathological and control brain.

### Effects of alcoholism on *PDYN* expression; influence of cell composition

**Fig. 2:**
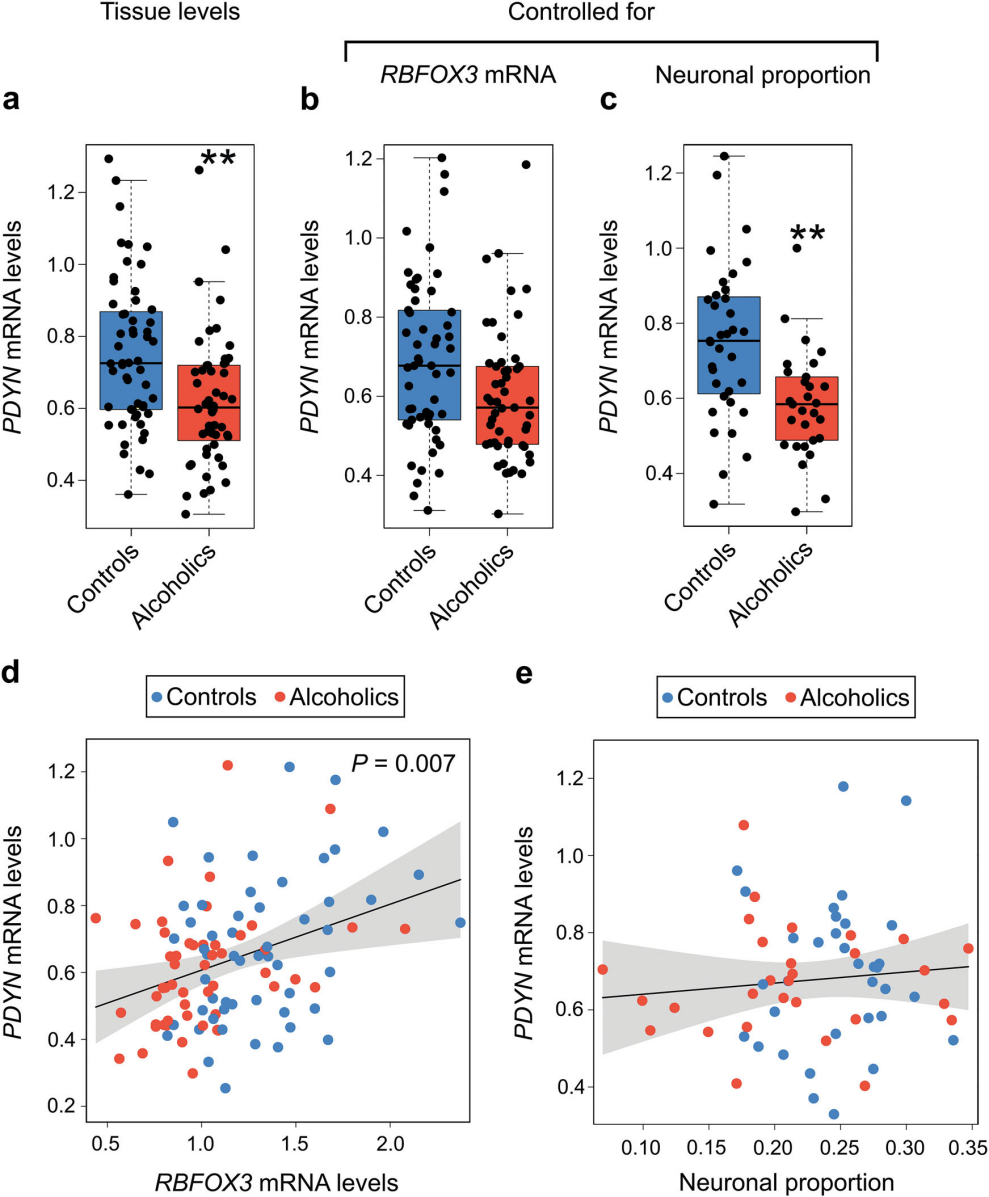
Effects of alcoholism on the tissue *PDYN* expression levels, and the expression levels controlled for cell composition in human dlPFC. **a** *PDYN* mRNA levels were significantly lower in alcoholics (*P* = 0.004). **b** Expression of *PDYN* controlled for *RBFOX3* mRNA levels differed between the two groups with a trend (*P* = 0.086). **c** Expression of *PDYN* controlled for neuronal proportion was significantly lower in alcoholics (*P* = 0.006). **d** Relationship between *PDYN* and *RBFOX3* mRNA levels; correlation was positive and significant (*P* = 0.007). **e** *PDYN* and neuronal proportion did not significantly correlate (*P* = 0.453). Data from the cohort of 55 controls and 53 alcoholics (**a**, **b**, **d**), or the cohort of 35 controls and 30 alcoholics (**c**, **e**) were analyzed via linear regression. mRNA levels are shown in arbitrary units. In box plots middle line is the median, box spans the interquartile range (IQR), and whiskers extend 1.5 × IQR from box limits. Lines and shading represent the estimated slopes and 95% confidence intervals, respectively. **P < 0.01 by ordinary bootstrap with 5 × 10^5^ nonparametric resampling of cases

We next examined whether the tissue *PDYN* expression levels calculated as the ratio to reference genes were affected by alcoholism. *PDYN* mRNA levels in alcoholics compared to controls were found to be significantly lower, 1.24-fold (main effect of alcoholism, mean and 95% CI estimated by bootstrap resampling, −0.133 [−0.220, −0.041], *P* = 0.004; Fig. 2a). When adjusted for the levels of *RBFOX3* mRNA, a neuronal marker, or for cell composition, *PDYN* mRNA levels in alcoholics compared with controls were lower, respectively, with a trend (−0.088 [−0.183, 0.019], *P* = 0.086; 1.15-fold; Fig. 2b), or significantly (−0.166 [−0.285, −0.051], *P* = 0.006; 1.29-fold; Fig. 2c). Inclusion of smoking status as covariate in statistical models did not affect significance of differences identified in dlPFC (for tissue levels: −0.122 [−0.217, −0.012], *P* = 0.019) and when corrected for cell composition (−0.146 [−0.265, −0.025], *P* = 0.019). No effects of severity of alcohol use disorder (*P* = 0.834), mean lifetime alcohol consumption (*P* = 0.127), or lifetime alcohol consumption (*P* = 0.581) on *PDYN* expression were found. *PDYN* mRNA significantly correlated with *RBFOX3* mRNA levels (main effect of *RBFOX3* mRNA, mean and 95% CI, 0.197 [0.068, 0.340], *P* = 0.007; Fig. 2d), but not with neuronal proportion (Fig. 2e). These results suggest that *PDYN* transcription is downregulated in dlPFC in alcoholics. This decrease does not depend on changes in cell composition, and consistently *PDYN* does not correlate with neuronal proportion. However, *PDYN* expression may be associated with that of neuronal marker NeuN (*RBFOX3*).

### Effects of alcoholism on KOR expression; influence of cell composition

**Fig. 3:**
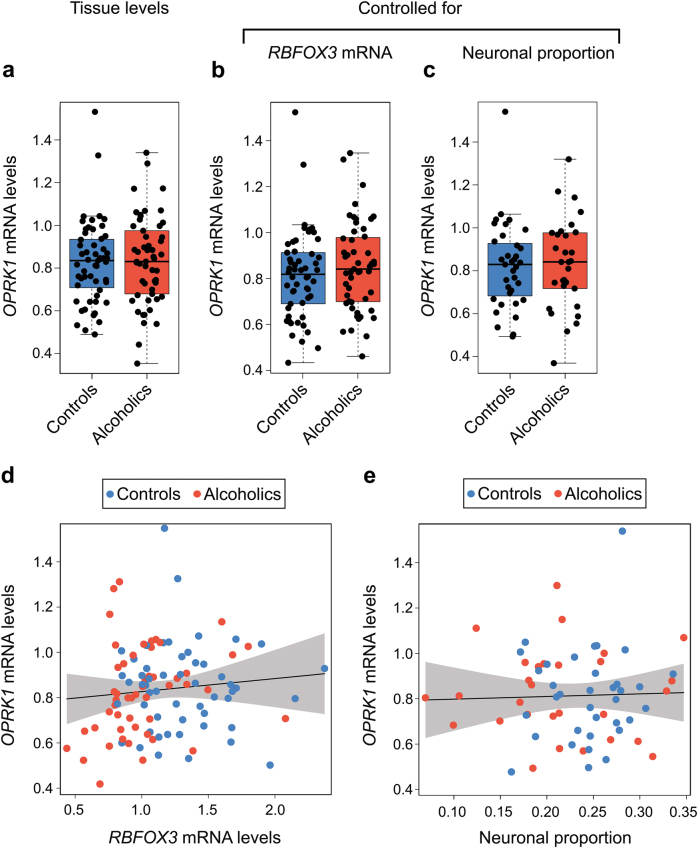
Effects of alcoholism on the tissue KOR (*OPRK1*) expression levels, and the expression levels controlled for cell composition in human dlPFC. **a** *OPRK1* mRNA levels were not altered in alcoholics (*P* = 0.802). **b** Expression of *OPRK1* controlled for *RBFOX3* mRNA levels was not different between the two groups (*P* = 0.403). **c** Expression of *PDYN* controlled for neuronal proportion was not significantly different in alcoholics (*P* = 0.863). **d** Relationship between *OPRK1* and *RBFOX3* mRNA levels; no significant correlation was found (*P* = 0.454). **e** Relationship between *OPRK1* and neuronal proportion; no significant correlation was found (*P* = 0.792). Data from the cohort of 55 controls and 53 alcoholics (**a**, **b**, **d**) or the cohort of 35 controls and 30 alcoholics (**c**, **e**) were analyzed via linear regression. mRNA levels are shown in arbitrary units. In box plots middle line is the median, box spans the interquartile range (IQR), and whiskers extend 1.5 × IQR from box limits. Lines and shading represent the estimated slopes and 95% confidence intervals, respectively. *P-*values were estimated by ordinary bootstrap with 5 × 10^5^ nonparametric resampling of cases

No significant effects of alcoholism on KOR (*OPRK1*) mRNA were evident for the tissue levels (Fig. 3a) and also the levels adjusted for *RBFOX3* expression levels (Fig. 3b) and for cell composition (Fig. 3c). Inclusion of smoking status as covariate revealed no differences between the subject groups. No effects of severity of alcohol use disorder (*P* = 0.768), mean lifetime alcohol consumption (*P* = 0.415), or lifetime alcohol consumption (*P* = 0.479) on *OPRK1* expression were found. No significant correlation was found between *OPRK1* and *RBFOX3* mRNA levels (Fig. 3d), and *OPRK1* mRNA levels and neuronal proportion (Fig. 3e). These data imply that the number of neurons producing *PDYN* or KOR is not proportional to the total number of neurons in dlPFC, and that the *PDYN* and KOR expressing neuronal subtypes and total neuronal population may be differentially affected by alcoholism.

### Correlation between *PDYN* and KOR

**Fig. 4:**
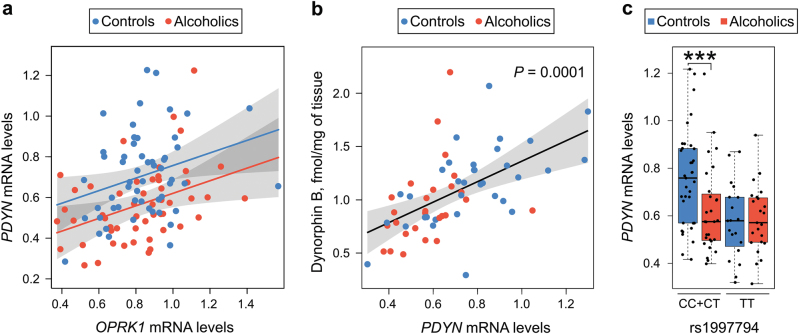
Relationship between *PDYN* and KOR (*OPRK1*) mRNAs (**a**), and between *PDYN* mRNA and dynorphin B (**b**) in dlPFC of controls and alcoholics. Effects of *PDYN* promoter SNP rs1997794 genotype on the *PDYN* tissue expression levels in dlPFC of alcoholic and control subjects (**c**). **a** Linear regression model corrected for age, PMI, brain pH, and RQI revealed a trend for positive correlation between *PDYN* and *OPRK1* mRNAs in the combined controls and alcoholics group (*n* = 108; main effect of *OPRK1* mRNA; *P* *=* 0.061). Effects of alcoholism × *OPRK1* mRNA interaction on *PDYN* mRNA levels were not significant (*n* = 55 controls and 53 alcoholics; *P* = 0.998), demonstrating no difference in the slope of the regression lines between the two subject groups. **b** Linear regression model corrected for age, PMI, brain pH, and RQI revealed a positive correlation between *PDYN* mRNA and dynorphin B in the combined controls and alcoholics group (*n* = 68; main effect of *PDYN* mRNA; *P* = 0.0001). Effect of alcoholism on dynorphin B levels was not significant (*P* = 0.605). **c** Significant effect of alcoholism × genotype interaction on *PDYN* mRNA levels was found (*n* = 55 controls and 53 alcoholics; *P* = 0.016). *PDYN* mRNA levels were significantly 1.37-fold lower in alcoholics carrying C (combined CC and CT genotypes, *n* = 28) high-risk allele of rs1997794 (*P* = 0.0001) compared to control subjects with the same genotype (*n* = 35). mRNA levels are shown in arbitrary units, dynorphin B levels as fmol/mg of tissue. In box plots middle line is the median, box spans the interquartile range (IQR), and whiskers extend 1.5 × IQR from box limits. Lines and shading represent the estimated slopes and 95% confidence intervals, respectively. *P-*values were estimated by ordinary bootstrap with 5 × 10^5^ resampling of cases. ***P < 0.001 by post hoc *t*-test

The *PDYN* and *OPRK1* genes may be co-expressed (i.e., transcriptionally co-regulated) and their co-expression pattern may be affected upon transition from normal to alcoholic state. To address this hypothesis we compared the slopes of regression lines for *PDYN* and *OPRK1* mRNA levels between controls and alcoholics. *PDYN* and *OPRK1* mRNAs correlated with a trend (main effect of *OPRK1* mRNA, 0.309 [−0.003, 0.639], *P* = 0.061; Fig. 4a), while interaction effect of alcoholism on *PDYN*—KOR correlation was not significant. Thus, correlation between *PDYN* and *OPRK1* expression did not differ between alcoholics and controls; the slope of regression line was similar in alcoholics and controls. The effect of alcoholism was not sensitive to (i) changes in cell composition assessed using neuronal marker NeuN (*RBFOX3*) or neuronal proportion, and to (ii) smoking status. This suggests a low level of coordination in regulation of these two genes both in controls and alcoholics.

### Correlation between *PDYN mRNA* and dynorphin B, a mature peptide *PDYN* product

*PDYN* mRNA correlated with dynorphin B, the peptide product of this gene (main effect of *PDYN* mRNA, 0.960 [0.550, 1.400], *P* = 0.0001; Fig. 4b), while no differences in the level of this endogenous kappa-agonist were found between alcoholics and controls (main effect of alcoholism, 0.052 [−0.128, 0.280], *P* = 0.605).

### Effects of alcoholism-associated SNPs on *PDYN* and *OPRK1* expression in controls and alcoholics

The level of alcohol-dependent activation of endogenous opioid transmission might be genetically determined^54,55^. We therefore examined whether adaptive *PDYN* responses to alcohol may be modulated by *PDYN* SNPs associated with alcoholism including promoter SNP rs1997794, and SNPs rs6045819 and rs2235749 located in coding exon 4 and 3′ untranslated region (3′-UTR) of the gene^56,57^. *PDYN* promoter SNP (rs1997794) was also found to be associated with better episodic memory scores in elderly humans^22^, and may form non-canonical AP-1 binding site and influence gene expression in human brain^58^.

The Fisher’s exact test revealed no significant difference in distribution of *PDYN* promoter SNP rs1997794 genotypes (*P* = 0.329) between alcoholics and control subjects (CC and CT genotypes vs. TT genotype; subjects with the C, high-risk genotype were pooled); or other SNPs (rs6045819, *P* = 0.528; rs2235749, *P* = 0.679; rs6985606, *P* = 0.326).

Two-way ANCOVA with group (controls vs. alcoholics) and *PDYN* promoter SNP rs1997794 genotype as between factors revealed a significant effect of group × genotype interaction (−0.179 [−0.320, −0.030], *P* = 0.016). Main effect of genotype was significant (0.186 [0.089, 0.287], *P* = 0.0003), while main effect of alcoholism was not significant (−0.022 [−0.119, 0.077], *P* = 0.659). For the combined CC and CT genotypes post hoc *t-*test showed downregulation of *PDYN* (1.37-fold; *P* = 0.0002) in alcoholics (Fig. 4c). The effect of alcoholism × genotype interaction was not sensitive to changes in cell composition assessed using neuronal marker NeuN (*RBFOX3*) (−0.148 [−0.289, −0.0002], *P* = 0.044). No other SNPs tested significantly affected *PDYN* or *OPRK1* expression between controls and alcoholics.

## Discussion

The main finding of our study is downregulation of *PDYN* expression in dlPFC of alcoholics. DYNs have a role in learning and memory acquisition, while their elevated levels are deteriorating for cognitive processes^17–19^. Impairments of spatial learning and memory by ethanol exposure were found to be mediated through activation of the KOR^16^. KOR antagonists abrogated alcohol-induced pathological processes. In this framework, the decrease in *PDYN* expression in dlPFC may be interpreted as adaptation that may counteract cognitive decline developed over the years of heavy alcohol drinking and withdrawal.

Postmortem studies of *PDYN* and KOR (*OPRK1*) regulation in addicted human brain are limited in a number and mostly focused on the striatal subregions. *PDYN* was found to be downregulated in NAc core in heroin addicts^59^, while no changes in *PDYN* or DYN and KOR protein expression in NAc of cocaine addicts^60,61^ were revealed. The *PDYN* and KOR (*OPRK1*) gene expression and DYN levels were reported to be elevated in dorsal striatum of cocaine addicts^60,61^, while the *PDYN* mRNA levels were decreased in dorsal striatum in alcoholics^44^ and in caudate nucleus of cocaine addicts carrying *PDYN* SNP variant associated with cocaine/alcohol codependence^57^. The absence of consistency in these data is likely due to underpowered postmortem human brain analysis in some of these works. Sample size calculation for *PDYN* expression levels in alcoholics and controls demonstrated that analyses with *n* ≤ 30, including our pilot study with 14 alcoholics and 14 controls, which were not overlapping with the current sample, and which suggested modest elevation in *PDYN* expression in dlPFC of alcoholics^35^, are likely underpowered. “Medium” size effects^62^ may be detected with *n* = 90 or larger sample size. In this study, we aimed to detect “large” effects by ensuring final sample size *n* ≥ 90. Totally 108 subjects were analyzed that is likely the largest cohort of alcoholics and controls investigated to date. In addition, we applied a more stringent statistical analysis that included available confounding factors (age, PMI, brain pH, RQI, and smoking status). Alcoholism is associated with the decline in neuronal proportion in dlPFC^37–39^. Inclusion of changes in cell composition did not affect the significance of differences between the groups in *PDYN* expression, thus demonstrating that this is a robust phenomenon. Based on these issues we may conclude that the identified differences are characteristic of the analyzed population of alcoholics, who were males of European descent. Importantly, the in vitro study supports this notion by demonstrating a significant decrease in the expression of *PDYN* in human neuroblastoma SH-SY5Y cells following exposure to ethanol^63^.

This study focuses on changes in expression and co-expression levels of the *PDYN* and KOR genes in alcoholics. Statistical analysis did not reveal effects of cell composition, suggesting that the observed expression differences may be caused primarily by adaptations in transcriptional mechanisms. These transcriptional changes may have functional consequences contributing to the downregulation of the DYN/KOR circuits in dlPFC of addicted individuals. In dlPFC the absolute expression levels of *PDYN* were significantly lower compared to those of KOR (*OPRK1*). Thus, *PDYN* expression may be a limiting factor in the DYN/KOR signaling, while its downregulation suggests the diminished efficacy of the DYN/KOR signaling in dlPFC of human alcoholics.

Development of drug/alcohol dependence may be viewed as a maladaptive habit formation during transition from recreational to compulsive use that is associated with a diminishing cognitive control over drug seeking and taking behavior^27,29,64–66^. These processes are characterized by a shift from prefrontal cortical to striatal control over drug/alcohol use, and a progression from the ventral to dorsal striatum in the addicted brain^64,67^. This shift is associated with the downregulation of the DYN/KOR system in both dlPFC (present study), and caudate and putamen^44^. Whether the paralleled DYN/KOR adaptations in dlPFC and dorsal striatum in alcoholics is a part of molecular processes underlying transition to the addictive state is a subject for future studies.

Associations between alcohol dependence and nine SNPs in the *PDYN* promoter and 3′-UTR and five SNPs in intron 2 of *OPRK1* were previously reported^56^. We selected three *PDYN* SNPs (rs1997794 located in the promoter, rs6045819 in coding exon 4, and rs2235749 in 3′-UTR) and one *OPRK1* SNP (rs6985606 located in intron 2) with highest significance of association (with *P* < 0.01) for analysis. *PDYN* promoter SNP rs1997794 and also 3′-UTR SNP rs910080 (located in the haplotype block with rs2235749) were also found to be associated with better episodic memory scores in elderly humans^22^. *PDYN* promoter SNP rs1997794 associated with alcoholism^56,57^ may form non-canonical AP-1 binding site and influence gene expression in human brain^58,68^.

For *PDYN* promoter SNP rs1997794, a significant effect of group × genotype interaction and significant main effect of genotype were found. *PDYN* downregulation was significant in the subgroup of subjects carrying C, high-risk allele of *PDYN* SNP rs1997794. This finding corroborates previous findings for the putamen, where *PDYN* mRNA and Leu-enkephalin-Arg, a peptide derived from PDYN, were significantly downregulated in the same subgroup of subjects^44^. No other SNPs tested significantly affected *PDYN* or *OPRK1* expression between controls and alcoholics.

As limitations, the identified associations may be only applicable to males of European descent because no female subjects and no other ethnic groups were analyzed. Human subjects were paired between two groups according to age, PMI, and brain pH and all confounding factors (age, PMI, brain pH, RQI, smoking status) were included in the analysis (Supplementary Table 1). The postmortem human findings may be interpreted in two ways, either as molecular adaptations in DYN/KOR expression patterns caused by heavy alcohol consumption and withdrawal or as manifestation of inherited molecular differences between controls and alcoholics.

The important limitation of the study is the absence of experimental background for functional implementation of the molecular findings at both molecular/cellular and behavioral levels. While *PDYN* mRNA correlated well with dynorphin B, the peptide product of this gene, no differences in the level of this endogenous kappa-agonist were found between alcoholics and controls. The DYN levels are regulated though synthesis and trafficking of their protein precursor molecule, processing of this molecule to mature opioid peptides, and release and degradation of the peptides. Furthermore there is a mismatch in anatomical localization of mRNA and mature peptide products. This mismatch occurs due to trafficking of the protein precursor molecules along neuronal projections to axon terminals in other brain areas, where these molecules are processed to mature DYNs^47^. These processes may be affected in alcoholic brain and, while not destroying the correlation, may shadow less pronounced differences between alcoholics and controls. We did not analyze relationship between the adaptations in the DYN/KOR system and cognate status of the individuals under study due to the absence of available premortal data. The functional consequence of *PDYN* downregulation in human alcoholics could be explored by analyzing the binding potential of selective kappa-opioid ligands, the development of which is rapidly progressing^69^.

In summary, our findings support the notion that the DYN/KOR signaling is dysregulated in human alcoholics. Chronic effects of various addictive substances may cause similar area-nonspecific transcriptional adaptations—the common downstream molecular syndrome mediating the lasting nature of the addictive state^66^. Downregulation of DYNs in dlPFC (present study), and dorsal striatum of alcoholics^44^ and cocaine addicts^57^ may be a part of this general adaptive mechanism.

## Electronic supplementary material

Supplementary Table 1

Supplementary Figure 1

Supplementary Figure 1 legend
