# Supplementary material for: Downregulation of the neuronal opioid gene expression concomitantly with neuronal decline in dorsolateral prefrontal cortex of human alcoholics
Source: Transl Psychiatry. 2018 Jun 20;8:122. doi: 10.1038/s41398-017-0075-5 (PMC6010434; doi:10.1038/s41398-017-0075-5)
Supplement: Supplementary file 1 — Supplementary Table 1 [file 41398_2017_75_MOESM1_ESM.docx]

**Supplementary Table 1** Demographic data and tissue characteristics of human subjects

| **Subject Id** | **Age** | **PMI** | **brain** | **RQI** | **Smoking status** |
| --- | --- | --- | --- | --- | --- |
|  | **(years)** | **(hours)** | **pH** |  |  |
| **Controls** |  |  |  |  |  |
| 1 | 60 | 13 | 6.59 | 8.6 | ? |
| 2 | 48 | 17 | 6.71 | 7.9 | No |
| 3 | 46 | 25 | 6.65 | 7.8 | ? |
| 4 | 58 | 12 | 6.46 | 7.8 | No |
| 5 | 50 | 19 | 6.26 | 8 | No |
| 6 | 48 | 24 | 6.73 | 8.2 | No |
| 7 | 43 | 66 | 6.2 | 7.1 | No |
| 8 | 57 | 18 | 6.6 | 8.1 | No |
| 9* | 60 | 25 | 6.7 | 6.2 | No |
| 10 | 63 | 24 | 6.94 | 8.2 | Yes |
| 11 | 73 | 48 | 6.8 | 8.2 | Yes |
| 12 | 64 | 9.5 | 6.94 | 9 | Yes |
| 13* | 73 | 51 | 6.82 | 5.9 | Yes |
| 14 | 53 | 27 | 6.64 | 7.4 | ? |
| 15 | 60 | 21.5 | 6.66 | 8.9 | No |
| 16* | 55 | 39 | 6.89 | 8.2 | No |
| 17* | 62 | 37.5 | 6.56 | 7.9 | ? |
| 18 | 47 | 38 | 6.74 | 8.8 | Yes |
| 19 | 68 | 45.5 | 6.12 | 5.9 | No |
| 20 | 50 | 30 | 6.37 | 8.3 | Yes |
| 21 | 59 | 40 | 6.53 | 6.8 | No |
| 22* | 56 | 19 | 6.9 | 8.3 | No |
| 23 | 55 | 12 | 6.39 | 8.9 | No |
| 24 | 73 | 38.5 | 6.28 | 8 | No |
| 25* | 62 | 46 | 6.95 | 8.1 | Yes |
| 26 | 36 | 34 | 6.67 | 8.3 | Yes |
| 27 | 50 | 40 | 6.87 | 8.3 | Yes |
| 28 | 54 | 28 | 6.38 | 8.7 | Yes |
| 29 | 58 | 28 | 5.92 | 3.3 | Yes |
| 30 | 69 | 52 | 6.95 | 8.5 | No |
| 31 | 53 | 16 | 6.5 | 8.8 | Yes |
| 32 | 37 | 14.5 | 6.46 | 8.5 | No |
| 33 | 57 | 18 | 6.39 | 8.5 | Yes |
| 34* | 50 | 34 | 6.77 | 8.7 | No |
| 35 | 59 | 29 | 6.61 | 8.8 | No |
| 36* | 37 | 24 | 6.7 | 7.7 | No |
| 37* | 51 | 35 | 7 | 8.4 | Yes |
| 38 | 59 | 15 | 6.54 | 8.4 | No |
| 39 | 47 | 27 | 6.66 | 8.7 | Yes |
| 40* | 64 | 29 | 6.55 | 7.7 | No |
| 41 | 61 | 22 | 6.41 | 8.8 | Yes |
| 42 | 40 | 27 | 6.79 | 8.9 | No |
| 43 | 61 | 30 | 6.69 | 8.8 | No |
| 44* | 59 | 28.5 | 6.81 | 8.7 | Yes |
| 45 | 40 | 59 | 6.93 | 8.9 | No |
| 46 | 64 | 30 | 6.82 | 8.1 | Yes |
| 47* | 48 | 50 | 6.68 | 8.6 | No |
| 48* | 46 | 26.5 | 6.68 | 8.7 | No |
| 49* | 49 | 22 | 6.88 | 9 | Yes |
| 50* | 59 | 49 | 6.86 | 8.1 | No |
| 51* | 55 | 23.5 | 6.88 | 9 | No |
| 52* | 39 | 22 | 6.49 | 8.6 | Yes |
| 53* | 50 | 29 | 6.82 | 8.4 | No |
| 54* | 57 | 37 | 6.49 | 8.3 | No |
| 55* | 55 | 25 | 6.08 | 7.1 | Yes |
| **Alcoholics** |  |  |  |  |  |
| 1 | 66 | 11.5 | 6.14 | 5.7 | Yes |
| 2* | 70 | 32 | 6.05 | 6.9 | ? |
| 3 | 54 | 17 | 6.41 | 7.7 | Yes |
| 4* | 51 | 27 | 5.58 | 5.6 | Yes |
| 5 | 50 | 24 | 6.59 | 7.3 | Yes |
| 6* | 52 | 35 | 6.04 | 6.9 | ? |
| 7 | 37 | 17 | 6.33 | 7.6 | No |
| 8 | 70 | 19 | 6.34 | 5.7 | Yes |
| 9 | 50 | 17 | 6.3 | 6.3 | ? |
| 10* | 51 | 46 | 6.35 | 6.4 | Yes |
| 11 | 67 | 48 | 6.4 | 7.9 | Yes |
| 12 | 70 | 62 | 6.82 | 7.7 | Yes |
| 13 | 42 | 41 | 6.5 | 7.8 | No |
| 14 | 58 | 20 | 6.64 | 7.9 | Yes |
| 15 | 43 | 29 | 6.29 | 6.7 | No |
| 16* | 57 | 43 | 6.46 | 8.2 | Yes |
| 17* | 60 | 16.5 | 6.48 | 9 | Yes |
| 18 | 73 | 43.5 | 6.59 | 7.1 | No |
| 19 | 58 | 21.5 | 6.65 | 8.7 | Yes |
| 20 | 63 | 25.5 | 6.21 | 3.5 | Yes |
| 21 | 73 | 19 | 6.84 | 8.3 | Yes |
| 22 | 54 | 27 | 6.16 | 6.4 | Yes |
| 23* | 40 | 40 | 6.42 | 8.3 | Yes |
| 24 | 65 | 14.5 | 6.79 | 9.1 | No |
| 25 | 55 | 48 | 7.02 | 7.5 | Yes |
| 26 | 64 | 39 | 6.76 | 8.5 | Yes |
| 27 | 55 | 17 | 6.85 | 9 | No |
| 28 | 59 | 35 | 6.57 | 5.6 | Yes |
| 29 | 61 | 27.5 | 5.87 | 5.6 | Yes |
| 30* | 56 | 65 | 6.47 | 6.2 | Yes |
| 31 | 58 | 44.5 | 6.47 | 8.1 | Yes |
| 32 | 65 | 72 | 6.88 | 7.1 | Yes |
| 33 | 69 | 22 | 5.82 | 5.7 | Yes |
| 34 | 43 | 33 | 6.57 | 8.4 | Yes |
| 35 | 61 | 59 | 6.57 | 8.1 | Yes |
| 36 | 49 | 44 | 6.41 | 8.5 | Yes |
| 37* | 62 | 40 | 6.59 | 7.5 | Yes |
| 38 | 63 | 28 | 6.89 | 9.1 | Yes |
| 39 | 62 | 30.5 | 6.79 | 9 | Yes |
| 40* | 44 | 59 | 6.87 | 8.2 | Yes |
| 41* | 55 | 27.5 | 6.56 | 6.7 | Yes |
| 42* | 60 | 28 | 6.48 | 6.5 | Yes |
| 43* | 50 | 34.5 | 6.93 | 8.5 | No |
| 44* | 61 | 52 | 6.63 | 7.3 | Yes |
| 45* | 47 | 72 | 5.92 | 3.8 | No |
| 46* | 40 | 50.5 | 6.83 | 8.4 | No |
| 47* | 54 | 22.5 | 6.77 | 8.6 | No |
| 48* | 51 | 51.5 | 6.8 | 7.7 | Yes |
| 49* | 53 | 53 | 6.77 | 7.6 | Yes |
| 50* | 52 | 47.5 | 6.7 | 7.5 | Yes |
| 51* | 47 | 36 | 6.51 | 8.6 | Yes |
| 52* | 55 | 24 | 6.43 | 8.1 | Yes |
| 53* | 56 | 39.5 | 6.34 | 8.2 | Yes |

Subject Id, subject’s depersonalized identification number; PMI, postmortem interval; RQI, RNA quality indicator.

* neuronal proportions were not measured
