# Supplementary figures and images for: Downregulation of the neuronal opioid gene expression concomitantly with neuronal decline in dorsolateral prefrontal cortex of human alcoholics
Source: Transl Psychiatry. 2018 Jun 20;8:122. doi: 10.1038/s41398-017-0075-5 (PMC6010434; doi:10.1038/s41398-017-0075-5)

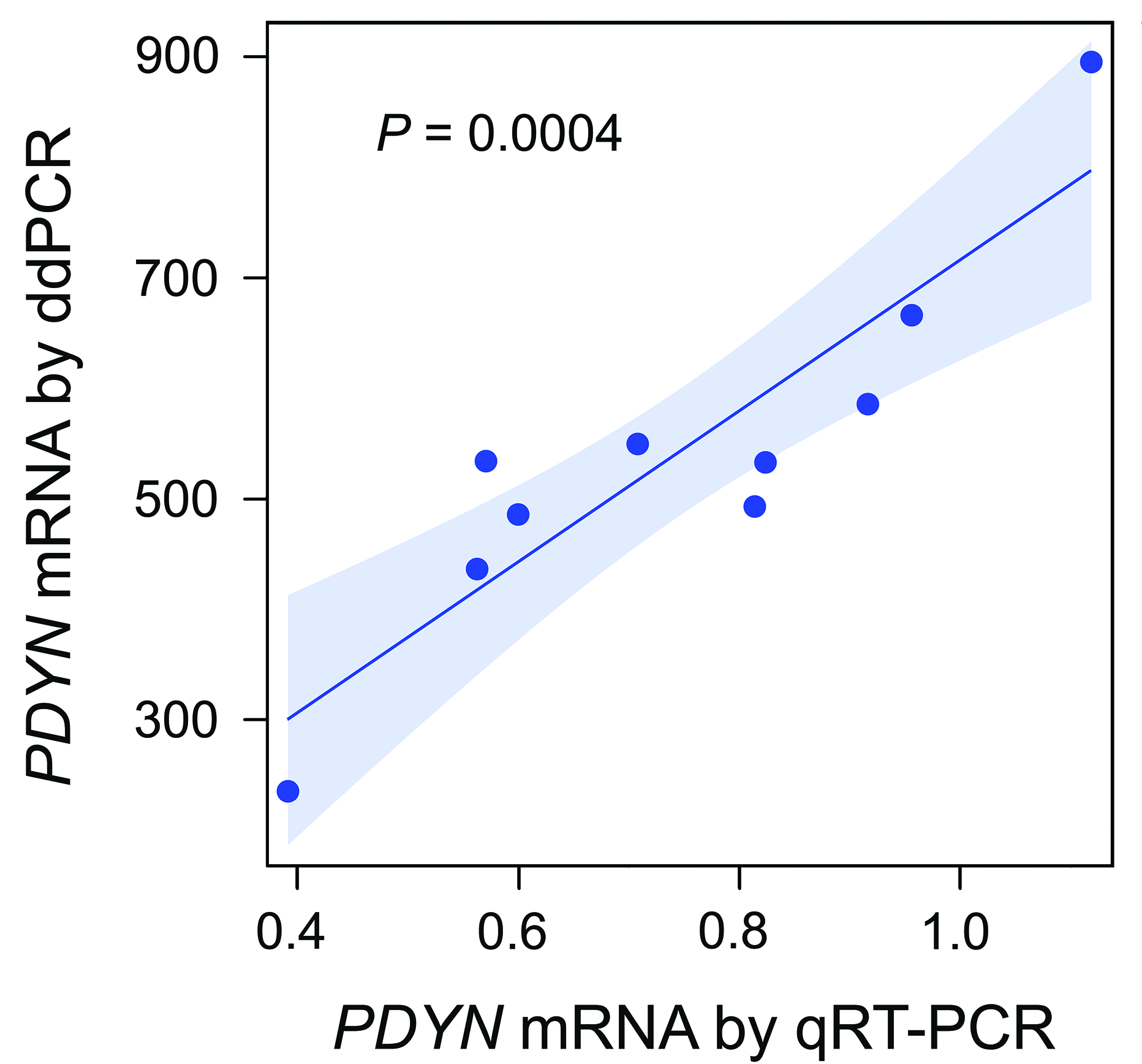

Supplement: Supplementary file 2 — Supplementary Figure 1 [file 41398_2017_75_MOESM2_ESM.tif]
