# Supplementary material for: Downregulation of the neuronal opioid gene expression concomitantly with neuronal decline in dorsolateral prefrontal cortex of human alcoholics
Source: Transl Psychiatry. 2018 Jun 20;8:122. doi: 10.1038/s41398-017-0075-5 (PMC6010434; doi:10.1038/s41398-017-0075-5)
Supplement: Supplementary file 3 — Supplementary Figure 1 legend [file 41398_2017_75_MOESM3_ESM.docx]

**Supplementary Figure 1.** Expression of the *PDYN* measured by ddPCR and qRT-PCR in human dlPFC (*n* = 10 control subjects). A significant correlation between the two methods was found (*P* = 0.0004). Absolute levels of *PDYN* mRNA measured by ddPCR using total tissue RNA presented as a number of mRNA copies/ng of total RNA, mRNA levels measured by qRT-PCR are shown in arbitrary units. Line and shading represent the estimated slope and 95% confidence interval, respectively.
